# Supplementary material for: The mechanism of NF-κB-TERT feedback regulation of granulosa cell apoptosis in PCOS rats
Source: PLoS One. 2024 Oct 25;19(10):e0312115. doi: 10.1371/journal.pone.0312115 (PMC11508119; doi:10.1371/journal.pone.0312115)
Supplement: S2 File — (ZIP) [file pone.0312115.s002.zip › The original data used to create the mechanism diagram in Figure 7.pptx]

## Slide 1
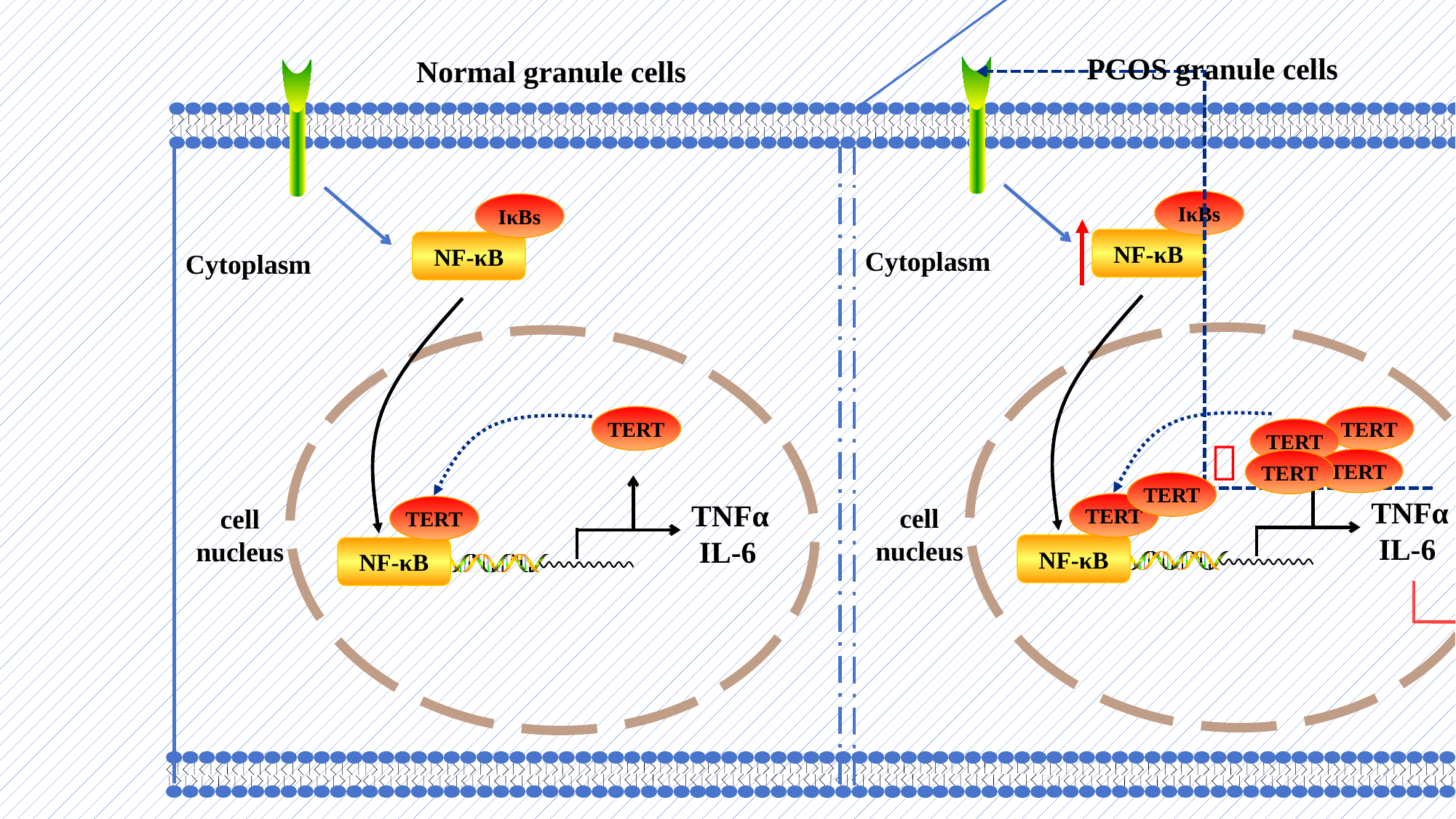

Ovary
Polycystic ovaries
Follicle
PCOS rat
Apoptotic cell
PCOS granule cells
Normal granule cells
IκBs
NF-κB
IκBs
NF-κB
Cytoplasm
Cytoplasm
TERT
TNFα
 IL-6
TERT
cell nucleus
NF-κB
TERT
TNFα
 IL-6
cell nucleus
TERT
NF-κB
Apoptosis
TERT
？
TERT
TERT
TERT
Inflammation
