# Supplementary material for: The mechanism of NF-κB-TERT feedback regulation of granulosa cell apoptosis in PCOS rats
Source: PLoS One. 2024 Oct 25;19(10):e0312115. doi: 10.1371/journal.pone.0312115 (PMC11508119; doi:10.1371/journal.pone.0312115)
Supplement: Attachment — Submitted filename: PONE-D-24-32182.docx [file pone.0312115.s003.docx]

The study makes a valuable contribution to understanding the role of NFKB-TERT pathway in PCOS-related ovarian dysfunction. While the authors have conducted a thorough investigation, several considerations merit attention prior to the manuscript's acceptance.

**Title:** The title might feel a bit technical. Authors are encouraged to streamline the title.

**Abstract:**

- Technical terms could be avoided.
- Brief context should be added.
- Lack of quantitative data in the abstract.

**Introduction:**

- Authors are encouraged to remove redundancies, and improve transition/flowing.
- Apoptosis molecular mechanism should be expanded.
- Clearly articulate the research hypothesis.

**Methodology:**

- Authors must mention randomization/blinding during allocation of rats into groups.
- The study uses a well-established method to induce PCOS. It would be beneficial to add a reference.
- Were all the rats equally affected? How did the researchers ensure consistency across the PCOS group.
- Authors should mention whether the hormone levels were assessed at specific times in the oestrous cycle.
- Western blot: how was the normalization performed (which protein)? Indicate the statistical method.
- IHC: indicate the quantification method.
- Cell viability assay: specify the positive and the negative control. Replace the “ctrl” by “control”: this applies on the whole document.

**Discussion:**

- Lack of clarity in linking telomere length and PCOS.
- The inconsistent reporting of telomerase activity is not efficiently explained.
- While NFKB is undoubtedly important, the discussion should consider other signalling pathways (MAPK, JAK/STAT..).
- Need for detailed exploration of the specific apoptotic pathway involving Bax, bcl-2, and caspase-3. How does the pathways interact with the inflammatory signalling?
- Authors are encouraged to integrate the clinical relevance of the findings (new diagnostic criteria or targeted therapy….).
- Improve the flow between the topics.
- How does targeting theses pathways could enhance the therapeutic strategies? Discuss the potential therapies.
- Address the limitations of the study.

**Conclusion:** Conclusion could be improved. Figure 7 could be transferred to the end of the discussion.
