# Supplementary material for: The mechanism of NF-κB-TERT feedback regulation of granulosa cell apoptosis in PCOS rats
Source: PLoS One. 2024 Oct 25;19(10):e0312115. doi: 10.1371/journal.pone.0312115 (PMC11508119; doi:10.1371/journal.pone.0312115)
Supplement: Attachment — Submitted filename: Manuscript.docx [file pone.0312115.s004.docx]

**The mechanism of NF-κB-TERT feedback regulation of** **granulosa cell apoptosis in PCOS rats with follicular development disorder**

**Haoxuan Xue^1#^, Zecheng Hu^2#^, Shun Liu^1^, Shun Zhang^3^, Wenqin Yang^1^, Jiasi Li^1^, Chulin Yan^1^, Jiaming Zhang^1*^, Jing Zhang^1*^, Xiaocan Lei^1*^**

Haoxuan Xue^1#^, (email: [1250971873@qq.com](mailto:1250971873@qq.com))

Zecheng Hu^2#^ , (email: [huzecheng@outlook.com](mailto:huzecheng@outlook.com ) )

Shun Liu^1^, (email: [liushun20232023@163.com](mailto:liushun20232023@163.com))

Shun Zhang^3^, (email: [huageshunzi@163.com](mailto: huageshunzi@163.com))

Wenqin Yang^1^, (email: [yangwenqin0901@163.com](mailto:yangwenqin0901@163.com))

Jiasi Li^1^, (email: [2124436394@qq.com](mailto:2124436394@qq.com))

Chulin Yan^1^, (email: [1823986254@qq.com](mailto:1823986254@qq.com))

Jiaming Zhang^1*^, (email: [zhangjia_ming123@163.com](mailto:zhangjia_ming123@163.com))

Jing Zhang^1*^, (email: [41314619@qq.com](mailto:41314619@qq.com))

Xiaocan Lei^1*^, (email: [liulincun@163.com](mailto:liulincun@163.com))

1. The First Affiliated Hospital, Gynecology&Obstetrics and Reproductive Medical Center, School of Basic Medical Sciences,Hengyang Medical School,University of South China, Hengyang, Hunan 421001, China
2. Department of Breast and Thyroid Surgery, The First Affiliated Hospital, Hengyang Medical School, University of South China, Hengyang, Hunan, 421001, China
3. Department of Reproductive Medical Center, The Affiliated Hospital of Guilin Medical University, Guilin 541001, China

^#^Authors contributed equally to this work.

*Corresponding Authors: Jiamin Zhang, The First Affiliated Hospital, Gynecology&Obstetrics and Reproductive Medical Center, School of Basic Medical Sciences,Hengyang Medical School, University of South China, Hengyang,Hunan 421001, China ,E-MAIL: [zhangjia_ming123@163.com](mailto:zhangjia_ming123@163.com)；
Jing Zhang, The First Affiliated Hospital ,Gynecology&Obstetrics and Reproductive Medical Center ,School of Basic Medical Sciences, Hengyang Medical School, University of South China,Hengyang, Hunan 42100, China, E-MAIL: [41314619@qq.com](mailto:41314619@qq.com);

**ABSTRACT**

Polycystic ovary syndrome (PCOS) remains a significant endocrine disorder impacting female reproductive health. This study elucidates the role of Nuclear factor kappa-B (NF-κB)-Telomerase reverse transcriptase (TERT) feedback in PCOS granulosa cell apoptosis. Using letrozole and a high-fat diet, a PCOS rat model was established, along with an LPS-treated KGN cell inflammation model was established. NF-κB and TERT inhibitors (BAY 11-7082 and BIBR1532) were then administered to LPS-induced KGN cells. PCOS rats displayed disrupted estrous cycles, increased weight, elevated serum testosterone, cystic follicles, granulosa cell layer thinning, and reduced corpora lutea count. In PCOS rat ovaries, NF-κB, Interleukin-6 (IL-6), Tumor Necrosis Factor α (TNF-α), TERT, Bax, and Caspase-3 exhibited notable upregulation, while Bcl-2 decreased, with telomere elongation. Correlation analysis showed significant associations among NF-κB-related inflammatory factors, TERT, and apoptotic factors, positively correlating with Bax and Caspase-3, and negatively with Bcl-2. LPS-treated KGN cells demonstrated increased expression of inflammatory and pro-apoptotic factors, later restored post-treatment with NF-κB and TERT inhibitors. In conclusion, TERT may induce granulosa cell apoptosis by participating in the regulation of the NF-κB signaling pathway, thereby mediating the chronic inflammatory response of PCOS through downstream inflammatory factors IL-6 and TNF-α.

**Keywords:** PCOS; Apoptosis; granulosa cells; NF-κB; hTERT

**INTRODUCTION**

PCOS is a common and complex endocrine, metabolic and psychological disorder affecting 5–18% of women in reproductive age(1,2). The Rotterdam criteria (2003) are commonly employed for diagnosing PCOS, requiring the presence of at least two of the following three features: clinical and/or biochemical hyperandrogenism, irregular ovulation or anovulation, and polycystic ovarian morphology (PCOM)(1,3). The cause of PCOS is multifaceted, including strong epigenetic and genetic influences, accompanied by hyperandrogenism, chronic inflammation, obesity, insulin resistance and abnormal lipid metabolism(4,5,5,6). Research indicates that PCOS is characterized by a proinflammatory state, with emerging evidence supporting the presence of chronic low-grade ovarian inflammation in affected women(7,8). Patients with PCOS have significantly increased levels of proinflammatory factors, such as TNF-α, IL-6 and so on(9–11). The pathway used to control TNF-α and IL-6 is through the Regulation of NF-κB(12). What’s more, It has been shown that the NF-κB plays an important role in the inflammatory state of patients with PCOS(7).

Telomeres are specific structures located at the ends of linear chromosomes in eukaryotes. The maintenance of telomere length is essential for the normal structure and function of telomeres and the integrity and stability of chromosome ends(13). Each cell division shortens the length of telomere 5'-TTAGG-3' sequence, which eventually leads to apoptosis or senescence(14).Telomerase is a ribonucleoprotein complex comprising TERT, telomerase RNA component (TERC), and telomerase-associated proteins (TEP).(15). TERC was used as a template for telomerase. TERT acts as a catalytic subunit to reverse transcribe telomeric DNA and maintain telomere length(16). The expression level of TERT is the main determinant of telomerase activity(17). The initiation of telomerase is a prerequisite for maintaining telomere length and genome integrity and achieving cell immortalization and malignant transformation(18). Most human somatic cells do not show telomerase activity, and a few cells with vigorous division, such as stem cells(19), tumor cells(18), and germ cells(20), express telomerase activity to varying degrees. The study included women aged 20 to 30 years, comprising 40 PCOS patients and 35 healthy controls for comparison. It was found that the telomere length of granulosa cells in PCOS group was significantly longer than that in Control group, while the telomere length of peripheral blood leukocytes was not different from that in Control group(21). Another study showed that 75 PCOS patients and 81 non-PCOS women were included in the study, and it was found that there was no significant difference in leukocyte telomere length between the Control group and PCOS patients. Interestingly, however, when comparing the telomere length of granulosa cells between Controls and PCOS patients, the telomere length of PCOS patients was significantly longer. The p values remained significant after adjusting for age and body mass index(22). Pedroso et al. proposed that the maintenance of telomere length in cumulus cells of PCOS patients might be attributed to elevated telomerase TERT activity(23).

It has been proved that telomerase can regulate NF-κB signaling pathways, resulting in the stimulation of TNF-α and IL-6(24), and telomerase activity in cumulus cells was higher in the PCOS group(23). TERT is a component of telomerase, and it interacts with NF-κB(25). With Given this, it is crucial to ascertain whether there is an association between NF-κB-related chronic low-grade inflammation and TERT expression in PCOS ovaries. Recent studies have focused on the function of granulosa cells (GCs), which play an important role in folliculogenesis and follicle maturation(26). Apoptosis has been observed in GCs of PCOS patients(27). The pro-apoptotic factors Bax and Caspase-3 and the anti-apoptotic factor Bcl-2 play a crucial role in regulating the growth and apoptosis of GCs during follicular development(27,28,28,29). Studies have confirmed that the chronic inflammatory response in PCOS patients can affect the changes in telomere length and telomerase activity(30), and chronic inflammation can cause continuous oxidative stress, leading to cell apoptosis(31). Because the expression of TERT directly reflects the level of telomerase activity(13), and the activation of NF-κB signaling pathway can cause the release of TNF-α and IL-6, it is still not clear whether TERT can mediate chronic inflammatory response and induce granulosa cell apoptosis by participating in the regulation of NF-κB signaling pathway. Therefore, this study will investigate the interaction between inflammation and telomeres and how this feedback influences granulosa cell apoptosis, ultimately leading to impaired follicular development in PCOS rats.

**MATERIALS & METHODS**

**Animals and Experiment Protocol**

Female Sprague Dawley (SD) rats (n =12, 5 weeks old, 172 ± 8g) were obtained from Hunan SJA Laboratory Animal Co.Ltd (Changsha, China). The rats were reared at 25°C, 12 h light/dark cycle, and enough food and water were provided for free access. After one week of adaptive feeding, the rats were divided into the control (Ctrl) group (n =6) and the PCOS group (n =6). The Ctrl group were supplied with standard pellets of feed for 30 days; the PCOS group were fed with a high-fat diet (HFD, consisting of 61.5% standard food, 12% lard, 5% sucrose, 5% milk powder, 5% peanut, 10% egg, 1% sesame oil, 0.5% salt) and letrozole (1 mg/kg/day, dissolved in 1% carboxymethylcellulose [CMC](32)for 30 days to establish the PCOS model. All rats were anesthetized with an intraperitoneal injection of 20% urethane (0.6 mL 100 g^-1^) before sacrifice. Body weight was determined at the end of modeling. Blood samples were collected after anesthetization. Ovaries were isolated, weighed, and the left ovaries of rats from each group was fixed in 4% paraformaldehyde overnight before being embedded in paraffin. The right ovaries of rats from each group were stored at −80 °C for molecular analysis. All animal experiments were performed in accordance with Regulations on the Administration of Laboratory Animals of University of South China (Revised in 2017) and approved by the Animal Ethics Committee of University of South China (No. SYXK2020-0002).

**Vaginal Smears and Estrous Cycle Determination**

Vaginal smears were taken daily between 11:00 am and 12:00 for 10 days before the end of modeling.(33) The estrous cycle stage was determined by observation of the vaginal smears using a light microscope (Zeiss,Germany). The stages of the estrous cycle were determined by identifying predominant cell types of cells present in rat vaginal smears(34–37): nucleated epithelial cells (proestrus); cornified cells (estrus); and mixed cells (nucleated, cornified, leucocytes) (metestrus); leucocytes (diestrus).

**Serum Hormone Measurement**

All rats were anesthetized with 20% urethane, and the whole blood sample was collected from the abdominal aorta of rats immediately. The serum was further isolated by centrifuging at 2000 rpm for 10 min at 4 °C, and stored at −80 °C for further determination. The levels of serum T, follicle stimulating hormone (FSH) and luteinizing hormone (LH) were determined with radioimmunoassay (Beijing North Institute of Biotechnology Co., Ltd., Beijing, China).

**Hematoxylin-eosin (H&E) staining**

The left ovaries of rats from each group were fixed in 4% paraformaldehyde overnight before being embedded in paraffin, and then were sequentially sectioned with 5 μM- slices for hematoxylin and eosin (H&E) staining in order to examine the pathological structural alterations of the ovary.

**Real-Time Quantitative PCR (RT-qPCR) Analysis**

Total RNA from ovary tissues and cells was extracted using TRIZOL reagent (15596026, Thermo Fisher Scientific, Shanghai, China) and cDNA was synthesized with TransScript One-Step gDNA Removal and cDNA Synthesis SuperMix Kit (TRAN, Beijing, China). Real-time PCR analyses were performed with the 2× Universal SYBR Green Fast qPCR Mix Kit (RK21203, ABclonal) and Applied Biosystems QuantStudio 3 (Thermo Fisher Scientific). GAPDH was used as the reference and The critical threshold cycle (Ct)(36) value was determined for each reaction, which was transformed into relative quantification data using the 2^−^^ΔΔCt^ method. The experimental primers were Human IL-6 (HQP009670, GeneCopoeia), Human TERT (HQP018018, GeneCopoeia) and Human TNF-α (HQP018141, GeneCopoeia).The primer sequences used for amplification are shown in **Table 1**.

**Western Blot Analysis**

Ovarian tissue and KGN cell lysates were extracted by RIPA lysis buffer (CWBIO, Beijing, China) and PMSF (Solarbio). The protein concentration was detected by a BCA Protein Assay Kit (CWBIO). Denatured proteins were separated by 10% SDS-PAGE, electrophoretically transferred onto polyvinylidene fluoride membranes (Meck Millipore, Merck & Co., Inc., NJ, USA). Then, transferred membranes were blocked by PBST (phosphate buffered saline [PBS] with 0.1% Tween-20) containing 5% skim milk for 2 h. The membranes were respectively incubated with antibodies against NF-κB p65 (1:1000 dilution, #8242, Cell Signaling Technology), IL-6 (1:1000 dilution, TD6087, Abmart Shanghai Co.,Ltd.), TNF-α (1:1000 dilution, PY19810, Abmart Shanghai Co.,Ltd.), TERT (1:1000 dilution, TD7129, Abmart Shanghai Co.,Ltd.), Bax (1:1000 dilution, T40051, Abmart Shanghai Co.,Ltd.), Bcl-2 (1:1000 dilution, 13-8800, Thermo Fisher Scientific, Shanghai, China), Caspase-3 (1:1000 dilution, #9662, Cell Signaling Technology) and β-Tubulin (1:5000 dilution, R20005, Abmart Shanghai Co.,Ltd.) overnight at 4 °C, followed by HRP-conjugated affinipure goat anti-mouse IgG (H+L) (1:5000 dilution, SA00001-1; Protein Tech Group Inc., USA) or goat anti-rabbit IgG (H+L) (1:5000 dilution, SA00001-2; Protein Tech Group Inc.) for 2h at room temperature. Finally, eECL (CW0049M, CWBIO) was added, and the Tanon-5500 Chemiluminescence Imaging System was used to detect the chemiluminescence of protein bands. Finally, blotting images were quantified using Image J analysis software (JAVA image processing program, NIH, Bethesda, USA).

**Immunohistochemistry (IHC)**

Samples of ovarian tissues was fixed in 4% paraformaldehyde overnight before being embedded in paraffin, and then were sequentially sectioned with 5 μM- slices. The endogenous peroxidase activity was blocked by 3% H_2_O_2_ for 30 min. The slices were put in the microwave in 0.1 M sodium citrate (pH 6.0) three times for antigen retrieval,

permeabilized with 1% T riton X-100 and PBST for 30 min, and blocked with 5% bovine serum albumin for 45 min. Subsequently, the slides were incubated in a humidity chamber at 4 ℃ overnight with primary antibody NF-κB p65 (1:400 dilution, #8242; Cell Signaling Technology), IL-6 (1:100 dilution, TD6087, Abmart Shanghai Co.,Ltd.), TNF-α (1:200 dilution, PY19810, Abmart Shanghai Co.,Ltd.), TERT (1:100 dilution, TD7129, Abmart Shanghai Co.,Ltd.), Bax (1:100 dilution, T40051, Abmart Shanghai Co.,Ltd.), Bcl-2 (1:200 dilution, 13-8800, Thermo Fisher Scientific, Shanghai, China), Caspase-3 (1:100 dilution, #9662, Cell Signaling Technology). After washing with PBST , the slices were incubated with Biotin-SP-conjugated Affinipure Rabbit Anti-Goat IgG(H+L) (1:200, SA00004–4; Protein Tech Group Inc.) at room temperature and 37 °C for 45 min. The slices were then incubated with HRP-conjugated Streptavidin (1:200, SA00001–0; Protein Tech Group Inc.) at 37 °C for 45 min. The slides were finally treated with 3,3-diamino-benzidine (DAB) (20× Metal Enhanced DAB Substrate Kit; Solarbio) to allow the brown staining of the positive cases. PBST was used as a negative control. Finally, the stained sections were observed and photographed under a light microscope (BX43, Olympus).

**Measurement of ovarian telomere length**

Total RNA was extracted from ovarian tissue using TRIZOL reagent and reverse transcribed into cDNA. DNA was then used as a template for Rt-PCR analysis. The PCR reaction system consisted of 5 uL of 2×ChamQ SYBR qPCR Master Mix, 1 μL of primer mix, 1 uL of template DNA, and 3 uL of ddH2O. The reaction conditions for telomere gene Tel and reference gene 36B4 were basically the same, with 32 cycles for telomere gene and 40 cycles for reference gene, and melting curve analysis was performed after PCR reaction. Telomere length was analyzed by relative quantification method: Ct values of target gene and reference gene were obtained, ΔCt=Ct^Tel_^Ct^36B4^, telomere/single copy reference ratio (T/S) was calculated: 2^-Ct^= (2^CtTel^/2^Ct36B4^)^-1^, the relative T/S ratio 2^-ΔΔCt^=2^-(ΔCt1-ΔCt2)^, this ratio corresponds to the relative telomere length of the samples, where ΔCt1 is the T/S of each sample and ΔCt2 is the T/S of the reference sample. The primer sequences used for amplification are shown in **Table 2**.

**Cell Viability Assay**

Cell viability was measured by Cell Counting Kit-8 Solution Reagent (CCK-8, Beyotime, China). Briefly, cells were seeded in 96-well plates and cultured with various concentrations of BAY 11-7082(0,5,10,5 and 20μM) and BIBR1532(0,10,20,40 and 80μM). Cell viability was examined by following standard procedures. Experiments were performed in triplicate.

**Cell Culture and Treatment**

Human ovarian granulosa-like tumor cell line KGN, which originated from a stage Ⅲ invasive ovarian granulosa cell carcinoma in a 63-year-old woman, this cell line in review was considered as a model for understanding the regulation of steroidogenesis, cell growth, and apoptosis in human granulosa cells. In this study, KGN cells were purchased from Zhejiang Meisen Cell Technology Co., LTD (CTCC-003-0105). KGN cells were cultured with Dulbecco’s Modified Eagle’s Medium-high glucose (DMEM, Sigma, USA) supplemented with 10% Fetal Bovine Serum (FBS, Invitrogen 115 Gibco, USA) and maintained in an atmosphere of 5% CO2 at 37 °C. KGN cells were plated in 6 cm plates at 106 per well. After starving for 24 h, the cells were treated without or with LPS (100 μg/mL) (I2643; Sigma, USA) , BAY 11-7082 (10 μg/mL) ([HY-13453](https://www.medchemexpress.cn/BAY-11-7082.html), MCE, USA) , and C(40 μg/mL) ( HY-17353 , MCE, USA) for 24 h.

**Flow cytometry was employed to detect cell apoptosis**

KGN cells from drug-treated groups were digested using trypsin without EDTA (Biosharp, China). After terminating digestion, cell suspensions were collected in centrifuge tubes and centrifuged at 800 rpm for 5 minutes at room temperature, and the supernatant was discarded. The cells were then washed with pre-chilled 1×PBS (4°C) and centrifuged at 800 rpm for 5 minutes. FITC-conjugated Annexin-V apoptosis detection kit (556547, Becton Dickinson, USA) was used to label KGN cells from each group for subsequent flow cytometry analysis.

**Statistical Analysis**

Data were analyzed using GraphPad Prism 8.0 (GraphPad Software, CA, USA) and presented as the mean ± standard deviation. Significant differences between/within groups were evaluated by the unpaired t-test or one-way ANOVA followed by Bonferroni ’s post-hoc test. Correlation between variables was determined by Pearson’s correlation coefficient. P value <0.05 was considered to be statistically significant.

**RESULTS**

**Letrozole and high-fat diet induced clinical PCOS-like changes in SD rats**

As mentioned above, the pathogenesis of PCOS has been studied from many angles, but remains unexplored the role produced by the NF-κB-TERT pathway in the pathogenesis of PCOS, so we aims to elucidate this aspect in the current study. Therefore, a PCOS rat model was established by the treatment of HFD and letrozole. As shown in **Figure 1A** and **Figure 1B**, the body weight and the ovarian weight of rats in the PCOS group was significantly increased than that of the Ctrl group. Compared with the Ctrl group, the serum levels of testosterone was significantly higher in PCOS group (**Figure 1C**).Estrous cycle disorder is one of the main characteristics of PCOS(38, 39, 40). The normal estrous cycle in rats averages 4–5 days, which is generally divided into four stages—Proestrus, Estrus, Metestrus, and Diestrus(34). As shown in **Figure 1D**-**E**, the Ctrl rats had a regular estrous cycle. Briefly, samples dominated by nucleated epithelial cells indicated the proestrus stage, and those with primarily cornified squamous epithelial cells indicated the estrus stage. The metestrus stage was indicated by mixed cells (nucleated, cornified, leucocytes), while the diestrus stage was dominated by leukocytes. Compared with Ctrl rats, the PCOS rats displayed disrupted estrous cycles, which comprised only the metestrus or diestrus phases. Appearance of representative ovaries. Compared with the Ctrl group, multiple follicles with cystic expansion presented vacuolated and disorganized structure (**Figure 1F**). Assessment of the ovarian morphology showed a significant decrease in the number of follicles and corpus luteum in PCOS rats compared with those in the controls, whereas antral follicles with cystic expansion were increased. The thickness of the granulosa cell layer was also reduced **(Figure 1G**-**H)**.These results proved that we successfully constructed the PCOS rat model.

**Letrozole and high-fat diet induced PCOS increased the expression of NF-κB-related inflammatory factors and TERT in ovaries**

Subsequently, to investigate the association between NF-κB-TERT feedback regulation and ovarian granulosa cell apoptosis in PCOS rats, we initially assessed the levels of NF-κB-related inflammatory factors in ovarian tissues from both groups to elucidate the link between chronic low-grade inflammation in the ovary and TERT expression. The qRT-PCR results demonstrated an upregulation of NF-κB, IL-6, TNF-α, and TERT in PCOS rats (**Figure 2A**). Western blot analysis further confirmed the elevated levels of NF-κB, IL-6, TNF-α, and TERT in the PCOS group compared to the Ctrl group (**Figure 2B-C**). Immunohistochemistry analysis also supported these findings by revealing increased expression of NF-κB, IL-6, TNF-α, and TERT in the PCOS group (**Figure 2D**). Our findings suggest that induction of PCOS through letrozole administration and a high-fat diet leads to enhanced expression of NF-κB-related inflammatory factors as well as TERT within the ovary. Additionally, telomere length was determined using qPCR analysis on ovarian granulosa cells from PCOS rats. utilized telomere-specific primers for this analysis. As depicted in **Figure 2E**, Compared with the Ctrl group, the telomere length of the cells in the ovaries increased by 1 to 2 times. These results indicate that the activity of inflammatory markers in ovarian cells of PCOS rats is increased and TERT function is increased.

**The increase of ovarian cell apoptosis in PCOS rats is positively correlated with NF-κb related inflammatory factors, TERT and apoptosis factors**

To further validate our hypothesis, we proceeded to investigate the expression of apoptotic factors at both mRNA and protein levels. In comparison with the Ctrl group, we observed a significant increase in the mRNA levels of Bax and caspase-3, along with a decrease in Bcl-2 mRNA level in GCs from the PCOS group (**Figure 3A**). Additionally, compared to the Ctrl group, GCs from the PCOS group exhibited elevated protein levels of Bax and caspase-3, while showing reduced levels of Bcl-2 (**Figure 3B-C**). Immunohistochemical staining analysis yielded consistent results (**Figure 3D**). Subsequently, we conducted an extensive analysis to explore any potential relationship between NF-κB-related inflammatory factors, TERT, and apoptotic factors in PCOS; aiming to elucidate whether chronic low-grade ovarian inflammation and up-regulation of TERT are associated with apoptosis. **Figure 3E** demonstrates a significant correlation among NF-κB-related inflammatory factors, TERT, and apoptotic factors. Specifically, Nf-κb-related inflammatory factors and TERT displayed positive correlations with pro-apoptotic markers such as Bax and Caspase-3; conversely exhibiting negative correlations with anti-apoptotic factor Bcl-2. These findings indicate that ovarian granulosa cells undergo apoptosis in PCOS rats, and underscore a positive correlation between NF-κB-related inflammatory factors, TERT, and apoptosis.

**After LPS stimulation, NF-κb-related inflammatory factors, hTERT expression and apoptosis of KGN cells increased, and there was a correlation among three**

The results of animal experiments demonstrated a significant correlation between NF-κB, its downstream inflammatory factors, and TERT with apoptosis-related factors in PCOS rats. To further investigate the relationship between NF-κB-TERT regulation and PCOS granulosa cell apoptosis, KGN cells were treated with LPS at a concentration of 1 μg/ml to establish an inflammatory cell model. This model was divided into two groups: Ctrl group and LPS group. qPCR assay was employed to assess changes in mRNA expression levels of NF-κB, its downstream inflammatory factors, hTERT, and apoptosis-related factors in both groups. In the LPS group, there was a significant upregulation of mRNA expression for NF-κB p65 and its downstream inflammatory factors IL-6, TNF-α, hTERT as well as pro-apoptotic factors Bax and Caspase-3 (**Figure 4A**). Conversely, the expression of anti-apoptotic factor Bcl-2 was significantly downregulated. Based on our previous animal experiments which revealed a significant correlation between NF-κB, its downstream inflammatory factors, TERT with apoptosis-related factors in PCOS rat ovaries; we found that NF-κB along with its downstream inflammatory factors were positively correlated with pro-apoptotic markers while negatively correlated with anti-apoptotic markers. To validate these findings from animal experiments further analysis was conducted to explore the association between NF-kappa B along with its downstream inflammatory mediators (IL-6, TNF-α), hTERT and apoptotic markers within KGN cells. As depicted in **Figure 4B** it is evident that there exists a significant correlation among NF-kappa B along with its downstream inflammatory mediators (IL6, TNF-α) as well as hTERT concerning apoptotic markers. Specifically, NF-κB p65, IL-6, TNF-α, and hTERT were positively associated with pro-apoptotic proteins such as Bax and Caspase-3, while exhibiting a negative association with the anti-apoptotic protein Bcl-2..These results indicated that LPS-induced KGN cells exhibited increased expression of inflammatory factors and apoptosis, and NF-κB-related inflammatory factors and TERT were positively correlated with apoptosis.

**Inhibition of NF-κB and TERT reduced the expression of NF-κB-related inflammatory factors and TERT in KGN cells treated with LPS**

To further elucidate the mechanism underlying NF-κB-TERT feedback regulation on apoptosis of ovarian granulosa cells in PCOS rats, we treated LPS-induced KGN cells with 10μg/ml BAY 11-7082 and 40 μg/ml BIBR1532, respectively. Experimental groups included Control (Ctrl), LPS (LPS induction only), LPS+NF-κB inhibitor (LPS induction + NF-κB inhibitor treatment), and LPS+TERT inhibitor (LPS induction + TERT inhibitor treatment). qPCR and Western Blot analyses were employed to assess the expression levels of NF-κB, its downstream inflammatory factors, and TERT in these four groups. Our results demonstrated a significant upregulation of NF-κB p65 along with its downstream inflammatory factors IL-6, TNF-α, and TERT in the LPS group compared to the Ctrl group. However, both the groups treated with NF-κB inhibitor BAY 11-7082 and TERT inhibitor BIBR1532 exhibited a significant downregulation of NF-κB p65 as well as its downstream inflammatory factors IL-6, TNF-α, and hTERT when compared to the LPS group (**Figure 5A-B**). To further investigate changes induced by LPS in KGN cells regarding inflammatory factors secretion, we quantified TNF-α and IL-6 levels in cell culture medium using ELISA assay (**Figure 5C**). Our findings revealed significantly increased levels of these inflammatory factors in the LPS group compared to both Ctrl and inhibitor groups.

**Inhibition of NF-κB and TERT reduced apoptosis in LPS-treated KGN cells**

Upon observing a significant correlation among inflammatory factors, TERT, and apoptotic factors in KGN cells following LPS induction, we initiated further investigations into the alterations of apoptosis-related factors in these cells upon treatment with the NF-κB inhibitor BAY 11-7082 and the TERT inhibitor BIBR1532. The expression of apoptosis-related factors was assessed using qPCR and Western Blot analysis across four cell groups (**Figure 6A-C**). Our findings revealed that compared to the Ctrl group, pro-apoptotic factors Bax and Caspase-3 were significantly up-regulated while anti-apoptotic factor Bcl-2 was significantly down-regulated in the LPS group. However, when compared to the LPS group, both NF-κB inhibitor BAY 11-7082 and TERT inhibitor BIBR1532 exhibited significant down-regulation of pro-apoptotic factors Bax and Caspase-3 along with a notable up-regulation of anti-apoptotic factor Bcl-2. Moreover, we employed Annexin V combined with propidium iodide labeled flow cytometry for apoptosis detection, followed by result analysis using FlowJo software. According to the results depicted in **Figure 6D**, the apoptosis rate of KGN cells induced by LPS significantly increased compared to the Ctrl group. However, the apoptosis rate of LPS-induced KGN cells treated with NF-κB and TERT inhibitors approached that of the Ctrl group. These results suggest that chronic low-grade inflammation in PCOS patients triggers a positive feedback regulation of the NF-κB-TERT pathway, which subsequently leads to the release of downstream inflammatory factors IL-6 and TNF-α, thereby increasing the expression levels of pro-apoptotic markers BAX and Caspase-3. At the same time, it decreases the expression level of anti-apoptotic marker Bcl-2, which eventually leads to the apoptosis of granulosa cells.

**DISCUSSION**

PCOS is characterized by the presence of stunted follicles in the enlarged ovaries of affected females, typically accompanied by menstrual irregularities, hyperandrogenism, metabolic abnormalities, and mental disorders(39). Currently, PCOS is the most common cause of female anovulation and infertility(40). PCOS is a multifaceted systemic disease that significantly impacts women's overall health and well-being throughout their lifespan. It not only affects the reproductive endocrinology of young women, but also involves multiple systems such as glucose and lipid metabolism, cardiovascular and skin. However, the mechanism of polycystic ovary syndrome is still unclear, so it is urgent to clarify the exact pathogenesis of PCOS, improve the diagnostic criteria of PCOS, and develop more targeted drugs.

In recent years, the role of chronic low-grade inflammation in the pathogenesis of PCOS has attracted increasing attention. Recent studies have shown that inflammatory processes are closely related to ovulation and play an important role in ovarian follicle dynamics. A large body of evidence has shown that hyperandrogenism in PCOS patients is closely related to inflammation-related genes such as TNF-α, Tnf receptor 2 (TNFR2) and IL-6(10). Moreover, numerous studies have identified a strong association between PCOS-related inflammatory responses and visceral adipose tissue(41). Abdominal obesity is common in PCOS patients. Visceral adipose tissue recruits immune cells by increasing the production and secretion of inflammatory factors monocyte chemoattractant proteins (MCP), leading to inflammatory response and maintaining the inflammatory state of adipocytes(42). Hyperglycemia associated with insulin resistance in PCOS patients can also induce inflammatory responses, leading to NF-κb activation and oxidative stress. Oxidative stress is the key link of chronic low-grade inflammation in PCOS, and is closely related to the expression of related pro-inflammatory factors(42). Contemporary biomedical research indicates that sustained activation of the inflammatory response in ovarian tissue plays a pivotal role in the reproductive endocrine dysfunction of PCOS (43). On the one hand, the release of inflammatory cytokines in ovarian tissue directly damages granulosa cells, which is not conducive to the development of follicles, and then leads to rare ovulation or anovulation. On the other hand, it stimulates androgen production, which further aggravates ovulation disorder and leads to systemic hyperandrogenism. Simultaneously, the activation of the inflammatory response can interfere with insulin signal transduction, reducing insulin sensitivity and leading to insulin resistance and compensatory hyperinsulinemia. In conclusion, PCOS is closely linked to chronic low-grade inflammation.

As shown in the present study, we examined the changes in the expression levels of NF-κB and its downstream target genes IL-6 and TNF-α in ovarian granulosa cells of PCOS rats. The results showed that compared with the Ctrl group, the expression levels of NF-κB P65 and its downstream inflammatory factors IL-6 and TNF-α in the ovarian tissue of PCOS group were significantly increased. In order to further explore the chronic inflammatory response in the granulosa cells of PCOS rats, we continued to construct the granulosa cell inflammation model by adding LPS, and continued to detect the expression changes of the above factors. The same results were found, the expression of the above factors in LPS group was significantly higher than that in Ctrl group. Molecular biological studies on PCOS suggest that the continuous activation of chronic microinflammation is one of the molecular mechanisms closely related to the occurrence and development of the disease(44).The above results verify that the ovarian granulosa cells of PCOS rats may have a chronic inflammatory response mediated by NF-κB.

Since both telomere length and telomerase activity are important factors affecting follicular development, it has been proposed that abnormalities in the telomere/telomerase system may be one of the pathogenic mechanisms of PCOS(23). However, the existing research results based on the relationship between PCOS and telomere/telomerase system are still controversial. Wei (22) et al. found that the telomere length of granulosa cells in PCOS patients was significantly longer than that in non-PCOS patients undergoing in vitro fertilization (IVF), however, there was no statistically significant difference in the telomere length of peripheral blood leukocytes between the two groups. Changes in telomerase activity between the two groups were not reported. In addition, the persistent oxidative stress present in PCOS patients can lead to decreased telomere stability, and proinflammatory factors and ROS may lead to shorter telomere length (45) and reduced telomerase activity(46). Pedros et al. (23) measured telomere length and telomerase activity in various cell types in ovaries of PCOS patients compared to controls with normal menstrual cycles. Women in both groups underwent in vitro fertilization with intracytoplasmic sperm injection (IVF/ICSI). It was found that the telomerase activity of cumulus granulosa cells in PCOS group was significantly higher than that in Ctrl group. However, there was no significant difference in telomere length between the two groups. Using a similar research design, Li(47) et al. found that the telomere length of granulosa cells in PCOS patients was shorter than that in IVF patients without PCOS, but there was no significant difference in telomerase activity between the two groups. However, Wang et al.(21) conducted a study on women aged 20-30 years and compared 40 PCOS patients with 35 non-PCOS women and found that the telomere length of granulosa cells in the PCOS group was significantly longer than that in the Ctrl group, and there was no difference in the telomere length of peripheral blood leukocytes between the two groups.

Telomerase is a holoenzyme composed of two basic components: telomerase reverse transcriptase (TERT) and telomerase RNA (TERC). TERT is a protein with reverse transcriptase activity that synthetes a hexamer sequence at the end of the chromosome and is highly expressed in renewing tissues. It represents the main component of the holoenzyme, without which there is no enzyme activity(48). TERT is a major determinant of telomerase activity, and its expression levels are consistent with telomerase activity. In this study, we investigated the expression of TERT, a key telomerase gene, in granulosa cells of PCOS rats. We found that the expression of TERT in the ovarian tissue of rats in the PCOS group was significantly upregulated compared with the Ctrl group. Therefore, we continued to verify at the cellular level, and we found that the expression of hTERT in granulosa cells was significantly up-regulated in the LPS group compared with the Ctrl group. Since LPS can induce the activation of NF-κB signaling pathway(49). Combined with our previous results, we found that the expression of NF-κB and its downstream inflammatory factors and TERT was increased in ovarian granulosa cells of PCOS rats, and the NF-κB signaling pathway and hTERT expression were increased upon LPS induction. We further treated KGN cells with NF-κB and TERT inhibitors, and the expression of inflammatory factors and hTERT decreased. To confirm our idea, we went on to examine telomere length in ovarian cells from PCOS rats and found that telomere length was significantly longer in PCOS than in Ctrl rats. Some studies have found that the telomere length of leukocytes in PCOS women is significantly shortened(22), due to the presence of telomerase activity in granulosa cells, but not in leukocytes, and its activity is an important factor in determining telomere length. In this study, the telomere length of granulosa cells in PCOS rats was significantly longer than that in Ctrl rats, which may be due to the higher TERT activity in granulosa cells of PCOS rats. We speculate that the increased expression of TERT in ovarian granulosa cells of PCOS patients is related to the chronic inflammatory response mediated by NF-κB, and there is an interaction between the two.

Apoptosis plays a key role in follicular development. granulosa cells are one of the main functional cells involved in the development of follicles, and some studies have shown that a variety of clinical manifestations of PCOS patients are closely related to granulosa cell dysfunction(50). Cataldo(51), Ding(52), Zheng (53) et al. found that the incidence of PCOS was closely related to the increase of apoptosis rate of ovarian granulosa cells. Bcl-2 family is involved in the regulation of cell apoptosis, among which the most representative is the anti-apoptotic factor Bcl-2 and the pro-apoptotic factor Bax. The imbalance of interaction between Bcl-2 and Bax is the main cause of cell apoptosis(28). The Caspase family also plays an important role in the molecular mechanisms that induce apoptosis, and it is the final convergence point of various apoptotic pathways(54).We examined the expression of Bax, Bcl-2 and Caspase-3 in the granulosa cells of PCOS rats. The results showed that Bax, Bcl-2 and Caspase-3 were expressed in the granulosa cells of PCOS rats. The expression of pro-apoptotic factors Bax and Caspase-3 in PCOS group was significantly up-regulated, and the expression of anti-apoptotic factor Bcl-2 was significantly down-regulated. Further experiments were carried out in KGN cells induced by LPS, The results showed that LPS induced significant up-regulation of Bax and Caspase-3 expression and down-regulation of Bcl-2 expression in granulosa cells. Furthermore, NF-κB inhibitor and TERT inhibitor were added to LPS-induced KGN cells, and the level of apoptosis was significantly decreased. By correlation analysis, the results showed that NF-κB and its downstream inflammatory factors and TERT were significantly correlated with apoptosis related factors in the ovarian cells of PCOS rats. At the cellular level, we obtained the same results. The above experimental results verified that the ovarian granulosa cells of PCOS had apoptosis, which was also the key cause of PCOS, and NF-κB and its downstream inflammatory factors and TERT were significantly correlated with apoptosis related factors.

**CONCLUSION**

In conclusion, this study observed a significant correlation between NF-κB-TERT and apoptosis-related factors in ovarian granulosa cells of PCOS rats. NF-κB-TERT exhibited positive correlations with pro-apoptotic factors and negative correlations with anti-apoptotic factors. TERT may participate in the regulation of NF-κB signaling pathway and activate the downstream inflammatory factors IL-6 and TNF-α to mediate the chronic inflammatory response of PCOS and induce the apoptosis of ovarian granulosa cells **(Figure 7)**.

**DATE AVAILABILITY STATEMENT**

The datasets used and/or analysed during the current study are available from the corresponding author on reasonable request.

**ETHICAL APPROVAL**

All animal experiments were approved by the Animal Ethics Committee of University of South China (No. SYXK2020-0002). The experiment was conducted in accordance with ARRIVE guidelines.

**AUTHORS’ CONTRIBUTIONS**

XHX, ZCH, JMZ and JZ designed the whole study. XHX, ZCH, JMZ, WQY, SL and SZ performed the experiments and analyzed the data. JZ, CLY, XCL, SZ and JSL performed the statistical analysis. JMZ and JZ wrote the manuscript. All authors approved the manuscript for submission.

**FUNDING**

This work was supported by the Scientific Research Foundation of Hunan Provincial Education Department (No. 20A434), The Natural Science Foundation of Guangxi in China (No. 2020GXNSFAA297097), The Natural Science Foundation of Hunan Province, China (No. 2020JJ5511 and 2024JJ9373), Scientific Research Foundation of Hunan Provincial Education Department (No. 22B0406), Health Research Foundation of Hunan Provincial Health Commission Scientific Researchand (No. W20243058), Foundation of Hengyang City science and technology innovation plan (No. 202330046470) and the Hunan Province Innovation and Entrepreneurship Training Program for College Students (No. S202310555229, S202310555214 and D202305162026050688).

**ACKNOWLEDGEMENTS**

Not applicable.

**CONFLICT OF INTEREST**

All of the authors declare no conflict of interest.

**REFERENCES**

1.	Escobar-Morreale HF. Polycystic ovary syndrome: definition, aetiology, diagnosis and treatment. Nat Rev Endocrinol. 2018 May;14(5):270–84.

2.	Joham AE, Norman RJ, Stener-Victorin E, Legro RS, Franks S, Moran LJ, et al. Polycystic ovary syndrome. Lancet Diabetes Endocrinol. 2022 Sep;10(9):668–80.

3.	The Rotterdam ESHRE/ASRM-sponsored PCOS consensus workshop group. Revised 2003 consensus on diagnostic criteria and long-term health risks related to polycystic ovary syndrome (PCOS). Hum Reprod. 2004 Jan 1;19(1):41–7.

4.	Akbarzadeh M, Naderi T, Dabbaghmanesh M. The glucose metabolism disorder and dyslipidemia among girls with different phenotype polycystic ovary syndrome. J Res Med Sci. 2019;24(1):72.

5.	Diamanti-Kandarakis E. Role of obesity and adiposity in polycystic ovary syndrome. Int J Obes. 2007 Nov;31(S2):S8–13.

6.	Wang J, Wu D, Guo H, Li M. Hyperandrogenemia and insulin resistance: The chief culprit of polycystic ovary syndrome. Life Sci. 2019 Nov;236:116940.

7.	González F, Rote NS, Minium J, Kirwan JP. Increased Activation of Nuclear Factor κB Triggers Inflammation and Insulin Resistance in Polycystic Ovary Syndrome. J Clin Endocrinol Metab. 2006 Apr 1;91(4):1508–12.

8.	Zhang X, Zhang C, Shen S, Xia YJ, Yi L, Gao Q, et al. Dehydroepiandrosterone induces ovarian and uterine hyperfibrosis in female rats. Hum Reprod. 2013 Nov 1;28(11):3074–85.

9.	Benson S, Janssen OE, Hahn S, Tan S, Dietz T, Mann K, et al. Obesity, depression, and chronic low-grade inflammation in women with polycystic ovary syndrome. Brain Behav Immun. 2008 Feb;22(2):177–84.

10.	Escobar-Morreale HF, Calvo RM, Villuendas G, Sancho J, San Millán JL. Association of Polymorphisms in the Interleukin 6 Receptor Complex with Obesity and Hyperandrogenism. Obes Res. 2003 Aug;11(8):987–96.

11.	Liu M, Gao J, Zhang Y, Li P, Wang H, Ren X, et al. Serum levels of TSP ‐1, NF ‐κB and TGF ‐β1 in polycystic ovarian syndrome ( PCOS ) patients in northern China suggest PCOS is associated with chronic inflammation. Clin Endocrinol (Oxf). 2015 Dec;83(6):913–22.

12.	Aggarwal BB, Shishodia S. Suppression of the Nuclear Factor-κB Activation Pathway by Spice-Derived Phytochemicals: Reasoning for Seasoning. Ann N Y Acad Sci. 2004 Dec;1030(1):434–41.

13.	Lewis KA, Wuttke DS. Telomerase and Telomere-Associated Proteins: Structural Insights into Mechanism and Evolution. Structure. 2012 Jan;20(1):28–39.

14.	Yu EY, Cheung NKV, Lue NF. Connecting telomere maintenance and regulation to the developmental origin and differentiation states of neuroblastoma tumor cells. J Hematol OncolJ Hematol Oncol. 2022 Aug 27;15(1):117.

15.	Shay JW. Role of Telomeres and Telomerase in Aging and Cancer. Cancer Discov. 2016 Jun 1;6(6):584–93.

16.	Lu W, Zhang Y, Liu D, Songyang Z, Wan M. Telomeres—structure, function, and regulation. Exp Cell Res. 2013 Jan;319(2):133–41.

17.	Ozturk S, Sozen B, Demir N. Telomere length and telomerase activity during oocyte maturation and early embryo development in mammalian species. Mol Hum Reprod. 2014 Jan 1;20(1):15–30.

18.	Shay JW, Wright WE. Senescence and immortalization: role of telomeres and telomerase. Carcinogenesis. 2005 May 1;26(5):867–74.

19.	Hiyama E, Hiyama K. Telomere and telomerase in stem cells. Br J Cancer. 2007 Apr;96(7):1020–4.

20.	Wright WE, Piatyszek MA, Rainey WE, Byrd W, Shay JW. Telomerase activity in human germline and embryonic tissues and cells. Dev Genet. 1996;18(2):173–9.

21.	Wang C, Shen F, Zhu Y, Fang Y, Lu S. Telomeric repeat-containing RNA (TERRA) related to polycystic ovary syndrome (PCOS). Clin Endocrinol (Oxf). 2017 Apr;86(4):552–9.

22.	Wei D, Xie J, Yin B, Hao H, Song X, Liu Q, et al. Significantly lengthened telomere in granulosa cells from women with polycystic ovarian syndrome (PCOS). J Assist Reprod Genet. 2017 Jul;34(7):861–6.

23.	Pedroso DCC, Santana VP, Donaires FS, Picinato MC, Giorgenon RC, Santana BA, et al. Telomere Length and Telomerase Activity in Immature Oocytes and Cumulus Cells of Women with Polycystic Ovary Syndrome. Reprod Sci. 2020 Jun;27(6):1293–303.

24.	Ghosh A, Saginc G, Leow SC, Khattar E, Shin EM, Yan TD, et al. Telomerase directly regulates NF-κB-dependent transcription. Nat Cell Biol. 2012 Dec;14(12):1270–81.

25.	Akiyama M, Hideshima T, Hayashi T, Tai YT, Mitsiades CS, Mitsiades N, et al. Nuclear Factor-␬B p65 Mediates Tumor Necrosis Factor ␣-induced Nuclear Translocation of Telomerase Reverse Transcriptase Protein. 2003;

26.	Fontana J, Martínková S, Petr J, Žalmanová T, Trnka J. Metabolic Cooperation in the Ovarian Follicle. Physiol Res. 2020 Feb 18;33–48.

27.	Gong Y, Luo S, Fan P, Zhu H, Li Y, Huang W. Growth hormone activates PI3K/Akt signaling and inhibits ROS accumulation and apoptosis in granulosa cells of patients with polycystic ovary syndrome. Reprod Biol Endocrinol. 2020 Dec;18(1):121.

28.	Chi XX, Zhang T, Chu XL, Zhen JL, Zhang DJ. The regulatory effect of Genistein on granulosa cell in ovary of rat with PCOS through Bcl-2 and Bax signaling pathways. J Vet Med Sci. 2018;80(8):1348–55.

29.	Hu CL, Cowan RG, Harman RM, Quirk SM. Cell Cycle Progression and Activation of Akt Kinase Are Required for Insulin-Like Growth Factor I-Mediated Suppression of Apoptosis in Granulosa Cells. Mol Endocrinol. 2004 Feb 1;18(2):326–38.

30.	Toupance S, Fattet AJ, Thornton SN, Benetos A, Guéant JL, Koscinski I. Ovarian Telomerase and Female Fertility. Biomedicines. 2021 Jul 20;9(7):842.

31.	Medzhitov R. Origin and physiological roles of inflammation. Nature. 2008 Jul 24;454(7203):428–35.

32.	Liang A, Huang L, Liu H, He W, Lei X, Li M, et al. Resveratrol Improves Follicular Development of PCOS Rats by Regulating the Glycolytic Pathway. Mol Nutr Food Res. 2021 Dec;65(24):2100457.

33.	Huang L, Liang A, Li T, Lei X, Chen X, Liao B, et al. Mogroside V Improves Follicular Development and Ovulation in Young-Adult PCOS Rats Induced by Letrozole and High-Fat Diet Through Promoting Glycolysis. Front Endocrinol. 2022 Mar 28;13:838204.

34.	Cora MC, Kooistra L, Travlos G. Vaginal Cytology of the Laboratory Rat and Mouse: Review and Criteria for the Staging of the Estrous Cycle Using Stained Vaginal Smears. Toxicol Pathol. 2015 Aug;43(6):776–93.

35.	Nallathambi A, Bhargavan R. Regulation of estrous cycle by *Cynodon dactylon* in letrozole induced polycystic ovarian syndrome in Wistars albino rats. Anat Cell Biol. 2019;52(4):511.

36.	Liang A, Zhang W, Wang Q, Huang L, Zhang J, Ma D, et al. Resveratrol regulates insulin resistance to improve the glycolytic pathway by activating SIRT2 in PCOS granulosa cells. Front Nutr. 2023 Jan 18;9:1019562.

37.	Maliqueo M, Benrick A, Stener-Victorin E. Rodent Models of Polycystic Ovary Syndrome: Phenotypic Presentation, Pathophysiology, and the Effects of Different Interventions. Semin Reprod Med. 2014 Apr 8;32(03):183–93.

38.	Arroyo P, Ho BS, Sau L, Kelley ST, Thackray VG. Letrozole treatment of pubertal female mice results in activational effects on reproduction, metabolism and the gut microbiome. Yu Y, editor. PLOS ONE. 2019 Sep 30;14(9):e0223274.

39.	Norman RJ, Dewailly D, Legro RS, Hickey TE. Polycystic ovary syndrome. The Lancet. 2007 Aug;370(9588):685–97.

40.	Hamilton-Fairley, Diana, Taylor, Alison, Braude, Peter. Anovulation. BMJ. 2003;

41.	Garruti G, Depalo R, Vita MG, Lorusso F, Giampetruzzi F, Damato AB, et al. Adipose tissue, metabolic syndrome and polycystic ovary syndrome: from pathophysiology to treatment. Reprod Biomed Online. 2009 Oct;19(4):552–63.

42.	Deligeoroglou E, Vrachnis N, Athanasopoulos N, Iliodromiti Z, Sifakis S, Iliodromiti S, et al. Mediators of chronic inflammation in polycystic ovarian syndrome. Gynecol Endocrinol. 2012 Dec;28(12):974–8.

43.	Dabravolski SA, Nikiforov NG, Eid AH, Nedosugova LV, Starodubova AV, Popkova TV, et al. Mitochondrial Dysfunction and Chronic Inflammation in Polycystic Ovary Syndrome. Int J Mol Sci. 2021 Apr 10;22(8):3923.

44.	He Z, Wang Y, Zhuan L, Li Y, Tang Z ouyin, Wu Z, et al. MIF-mediated NF-κB signaling pathway regulates the pathogenesis of polycystic ovary syndrome in rats. Cytokine. 2021 Oct;146:155632.

45.	Pedroso DCC, Miranda-Furtado CL, Kogure GS, Meola J, Okuka M, Silva C, et al. Inflammatory biomarkers and telomere length in women with polycystic ovary syndrome. Fertil Steril. 2015 Feb;103(2):542-547.e2.

46.	Li Y, Li R, Ouyang N, Dai K, Yuan P, Zheng L, et al. Investigating the impact of local inflammation on granulosa cells and follicular development in women with ovarian endometriosis. Fertil Steril. 2019 Nov;112(5):882-891.e1.

47.	Li Y, Deng B, Ouyang N, Yuan P, Zheng L, Wang W. Telomere length is short in PCOS and oral contraceptive does not affect the telomerase activity in granulosa cells of patients with PCOS. J Assist Reprod Genet. 2017 Jul;34(7):849–59.

48.	Rocca MS, Foresta C, Ferlin A. Telomere length: lights and shadows on their role in human reproduction. Biol Reprod [Internet]. 2018 Oct 1 [cited 2023 Sep 18]; Available from: https://academic.oup.com/biolreprod/advance-article/doi/10.1093/biolre/ioy208/5113454

49.	Liu Y, Liu H, Li Z, Fan H, Yan X, Liu X, et al. The Release of Peripheral Immune Inflammatory Cytokines Promote an Inflammatory Cascade in PCOS Patients via Altering the Follicular Microenvironment. Front Immunol. 2021 May 17;12:685724.

50.	Shalev E. The balance between MMP-9 and MMP-2 and their tissue inhibitor (TIMP)-1 in luteinized granulosa cells: comparison between women with PCOS and normal ovulatory women. Mol Hum Reprod. 2001 Apr 1;7(4):325–31.

51.	Cataldo NA, Dumesic DA, Goldsmith PC, Jaffe RB. Immunolocalization of Fas and Fas ligand in the ovaries of women with polycystic ovary syndrome: relationship to apoptosis*. Hum Reprod. 2000 Sep;15(9):1889–97.

52.	Ding L, Gao F, Zhang M, Yan W, Tang R, Zhang C, et al. Higher PDCD4 expression is associated with obesity, insulin resistance, lipid metabolism disorders, and granulosa cell apoptosis in polycystic ovary syndrome. Fertil Steril. 2016 May;105(5):1330-1337.e3.

53.	Zheng Q, Li Y, Zhang D, Cui X, Dai K, Yang Y, et al. ANP promotes proliferation and inhibits apoptosis of ovarian granulosa cells by NPRA/PGRMC1/EGFR complex and improves ovary functions of PCOS rats. Cell Death Dis. 2017 Oct 26;8(10):e3145–e3145.

54.	Bernard A, Chevrier S, Beltjens F, Dosset M, Viltard E, Lagrange A, et al. Cleaved Caspase-3 Transcriptionally Regulates Angiogenesis-Promoting Chemotherapy Resistance. Cancer Res. 2019 Dec 1;79(23):5958–70.

**Table1** Primer sequences used for the qRT-PCR analysis

| Gene ID | | Sequence | GeneBank Accession NO. |
| --- | --- | --- | --- |
| Rat NF-κB | Forward | AGCCACTGCCTTCCCTACTTC | [NM_001276711.2](https://www.ncbi.nlm.nih.gov/nuccore/NM_001276711.2) |
|  | Reverse | GGTCCTTAGCCCACTCCTTCTG |  |
| Rat TNF-α | Forward | GTCCCAACAAGGAGGAGAAGT | NM_012675.3 |
|  | Reverse | CTGGTATGAAATGGCAAATCG |  |
| Rat TERT | Forward | AGTGGTGAACTTCCCTGTGG | NM_053423.2 |
|  | Reverse | CAACCGCAAGACTGACAAGA |  |
| Rat Bax | Forward | GAGACACCTGAGCTGACCTT | XM_032913059 |
|  | Reverse | TCCATGTTGTTGTCCAGTTC |  |
| Rat Bcl-2 | Forward | AGTACCTGAACCGGCATCT | NM_016993 |
|  | Reverse | TCTTCAGAGACAGCCAGGA |  |
| Rat Caspase-3 | Forward | CCGGTTACTATTCCTGGAGA | XM_006253130 |
|  | Reverse | TAACACGAGTGAGGATGTGC |  |
| Rat GAPDH | Forward | TACACTGAGGACCAGGTTG | XM_032916238 |
|  | Reverse | CCCTGTTGCTGTAGCCATA |  |
| Homo NF-κB | Forward | CTGCCGGGATGGCTTCTAT | [NM_001165412.2](https://www.ncbi.nlm.nih.gov/nuccore/NM_001165412.2) |
|  | Reverse | CCGCTTCTTCACACACTGGAT |  |
| Homo Bax | Forward | GCGACTGATGTCCCTGTCT | NM_001291430 |
|  | Reverse | TGAGTGAGGCGGTGAGC |  |
| Homo Bcl-2 | Forward | CCCTGTGGATGACTGAGTACC | NM_000633 |
|  | Reverse | AGACAGCCAGGAGAAATCAAA |  |
| Homo Caspase-3 | Forward | CAGTGATGCTGTGCTATGAAT | NM_001354783 |
|  | Reverse | CAGATGCCTAAGTTCTTCCAC |  |
| Homo GAPDH | Forward | GAGTCCACTGGCGTCTTCAC | NM_001357943.2 |
|  | Reverse | GAGGCATTGCTGATGATCTTGAG |  |

**Table2** Primer sequences used for telomere length analysis

| Gene ID | | Sequence |
| --- | --- | --- |
| Rat Telomere | Forward | GTAATTGCGTAAGACTTAAAACC |
|  | Reverse | CCTAGAAATAAGAGGATTTAAACC |
| Rat 36B4 | Forward | ACTGGTCTAGGACCCGAGAAG |
|  | Reverse | TCAATGGTGCCTCTGGAGATT |

**Figure 1. Effects of letrozole and high-fat diet induction on body weight, ovarian weight, serum hormone levels, estrous cycle, and ovarian morphology.**

A: Body weight of each group (n=6 per group, day 60).

B: Bilateral total ovarian weight in each group.

C: Serum testosterone levels in each group (n=6 per group).

D: Cytological assessment of vaginal smears (n=5 per group, day 51-60).

E: Line chart of estrous cycle (n=5 per group, day 51-60).

F: Ovarian morphology of each group.

G: HE staining scan and magnification of ovarian sections in each group (n=6 per group, scale bar in upper is 500 μm). The lower panel show the higher magnification of the box area in the

upper panel respectively.

H: Counts of cystic follicles and corpus luteum (n=6 per group).

CL: corpus luteum; GC: granulosa cells. Significant differences between groups were indicated as ***P* < 0.01 and ****P* < 0.001.

**Figure 2. The levels of NF-κB related inflammatory factors, TERT expression and telomerase relative length are significantly increased in the ovarian tissue of PCOS rats.**

A: The expression changes of NF-κB, IL-6, TNF-α and TERT at mRNA level were detected by qRT-PCR (n=3 per group).

B-C: Western blotting was used to detect the protein expression of NF-κB, IL-6, TNF-α, and TERT, and ImageJ was used for quantitative analysis (n=3 per group).

D: Immunohistochemistry was used to detect the expression changes of NF-κB, IL-6, TNF-α and TERT at protein level (n=4 per group), the lowest panel shows the negative control.

E: Telomere length quantitative qRT-PCR results showed increased telomere length in the ovarian region of PCOS rats compared to controls (n=3 per group).

T/S ratio (telomere/single gene ratio); Data were presented as mean ± SEM. Significant differences were respectively presented as **P* < 0.05, ***P* < 0.01, ****P* < 0.001.

**Figure 3. Apoptosis is increased in the ovary of PCOS rats, and there is a correlation between NF-κB-related inflammatory factors, TERT and apoptosis factors.**

A: The expression changes of Bax, Bcl-2 and Caspase-3 at the mRNA level were detected by qRT-PCR (n=3 per group).

B-C: Western blotting was used to detect the changes in the expression of Bax, Bcl-2 and caspase-3 at the protein level, and ImageJ was used for quantitative analysis (n=3 per group).

D: The expression of Bax, Bcl-2 and caspase-3 at the protein level was detected by immunohistochemistry (n=4 per group)，the lowest panel shows the negative control.

E: Correlation analysis of inflammatory factors NF-κB, IL-6, TNF-α, telomerase reverse transcriptase TERT and apoptosis-related factors Bax, Bcl-2, Caspase-3 in ovarian tissue of rats in each group, Correlation between variables was determined by Pearson’s correlation coefficient. *P*<0.05 was considered to be statistically significant.

Data were presented as mean ± SEM. Significant differences were respectively presented as **P* < 0.05 and ***P* < 0.01.

**Figure 4. LPS treatment increases the expression of NF-κB-related inflammatory factors, hTERT and cell apoptosis in KGN cell and correlation between** **NF-κB-related inflammatory factors, TERT and cell apoptosis factors after LPS stimulation in KGN cell.**

A: The mRNA expression of NF-κB, IL-6, TNF-α, TERT, Bax, Bcl-2 and Caspase-3 in KGN cells in each group was detected by qRT-PCR.

B: The correlation between inflammatory factors (NF-κB, IL-6, TNF-α, TERT) and apoptosis-related factors (Bax, Bcl-2, Caspase-3) in KGN cells after LPS stimulation was analyzed. Pearson correlation coefficient was used to determine the correlation between variables. P<0.05 was considered statistically significant.

Data were presented as mean ± SEM. Significant differences were respectively presented as **P* < 0.05 and ***P* < 0.01.

**Figure 5. Changes in the expression of NF-κB-related inflammatory factors, TERT and telomerase activity** **in KGN cells in each group.**

A: The mRNA expression of NF-κB, IL-6, TNF-α and TERT in KGN cells of each group was detected by qRT-PCR.

B-C: Western blotting detected the expression of NF-κB, IL-6 and TNF-α proteins in KGN cells of each group, and ImageJ was used for quantitative analysis.

D: ELISA was used to detect the content of inflammatory factors (TNF-ɑ, IL-6) in the cell culture medium.

Data were presented as mean ± SEM. Significant differences were respectively presented as **P*< 0.05, ***P*< 0.01, ****P*＜0.001.

**Figure 6. Changes in the expression of apoptosis-related factors Bax, Bcl-2, and Caspase-3 in KGN cells in each group.**

A: The mRNA expression of Bax, Bcl-2 and Caspase-3 in KGN cells of each group was detected by qRT-PCR.

B-C: Western blotting detected the expression of Bax, Bcl-2 and Caspase-3 proteins in KGN cells of each group, and ImageJ was used for quantitative analysis.

D: The apoptosis of KGN cells in each group was detected by flow cytometry.

Data were presented as mean ± SEM. Significant differences were respectively presented as **P*< 0.05 and ****P*＜0.001.

**Figure 7. The NF-κB-TERT in PCOS ovarian feedback regulation mechanism of simple model.**

TERT may participate in regulating the NF-κB signaling pathway, activating downstream inflammatory factors IL-6 and TNF-α, mediating the chronic inflammatory response of PCOS, and inducing apoptosis of ovarian granulosa cells.
